# Noise exposure and auditory thresholds of military musicians: a follow up study

**DOI:** 10.1186/s12995-018-0196-7

**Published:** 2018-04-12

**Authors:** Reinhard Müller, Joachim Schneider

**Affiliations:** 1 Justus-Liebig-Universität Giessen, Aulweg 123, 35392 Giessen, Germany; 2 Institut und Poliklinik für Arbeits- und Sozialmedizin am Universitätsklinikum Giessen und Marburg, Aulweg 129, Giessen, 35392 Germany

**Keywords:** Military musicians, Audiometry, Hearing, Hearing loss, Noise exposure

## Abstract

**Background:**

Military musicians are working in a noisy environment with high sound exposure levels above the international standards. Aim of the current study is to find out, whether they develop the expected hearing impairments. Adherence to the regulations for prevention in musicians is more difficult than in other occupational fields.

**Methods:**

In an interval of 13.3 years, 36 out of 58 male military musicians of a German army music corps were subjected twice to an audiometric audit. There were no exclusion criteria apart from acute ENT infections (three musicians). These results were compared with one another and evaluated by means of statistical methods for relationships with several factors.

**Results:**

At frequencies below 3 kHz, the follow-up audiograms were up to 5 dB better than the preliminary examination. From 4 kHz up to 8 kHz the preliminary investigations showed less hearing impairment. Averaging all frequencies the improvement of hearing ability was around 1 dB. Above 1 kHz the average hearing of the right ear was up to 7 dB better than that of the left ear. Age-induced hearing loss was 3 to 8 dB lower than predicted by ISO standards over the entire frequency range. The side of the ear (right/left) and the frequency (3, 4, and 6 kHz) were significant (*p* < 0.05) in hearing loss, whereas the influence of the instrument and the acoustic traumata were not.

**Conclusion:**

Despite the high noise levels, the average hearing ability of the 36 military musicians during the investigation period only slightly deteriorated in the noise-sensitive frequencies (3, 5 and 6 kHz). Music may be less harmful than industrial noise, or the long-term auditory training of the musicians leads to a delayed presbycusis.

## Background

At the time of the old Israelites, trumpets are said to have been able to bring whole city walls to collapse, according to the relevant account in the Old Testament of the Bible, in Joshua 6. Even today, brass orchestras are able to produce a significant sound pressure, which may be felt in a larger radius. While lovers of military music appreciate these brass instruments and the sound, the usually excessive volume encounters refusal, especially with gentler natures. But not only military orchestras can produce high sound pressure levels but even ballet orchestras are suspected of hearing hazard [1].

The musicians as parts of the sound source themselves are more exposed to these sounds than the audience in some distance and should therefore take a greater risk to develop noise induced hearing loss. In order to determine these sonic loads, appropriate measurements were made in some studies. These relate predominantly to classical orchestras. Since the number of military musicians on the whole seems rather small, their hearing is rarely subject of published investigations. A search in PubMed with the search query: <“military musicians” AND “hearing”> provides only three matches. One of these articles [2] complains about the below-average poor health surveillance of military musicians without commenting on their hearing status, so that only two articles remain. Both were published in 2013. While a Brazilian study [3] reports an approximately 15-fold increased risk of hearing loss of military musicians compared to an unimpaired population, Patil et al. [4] reports no increased risk of hearing damage in British Army musicians compared to military administrative personnel. In 1981 Axelsson and Lindgren [5] already described in a larger study the hearing ability of orchestral musicians, that military music poses no increased risk of developing hearing damage compared to orchestral music. For most musicians with noticeable hearing damage (> 80%), a connection could be established with the music practice. Sound measurements in orchestras performed during rehearsals and public performances predominantly indicate level margins and average values which exceed the upper exposure action value of 85 dB(A) on daily shift [6]. McBride et al. [7] report up to 160% of the allowed daily dose on an average day with rehearsals and concert. According to ISO 1999 [8], therefore, the effects of sound exposure on the ear should be found. Mostly, however, lower levels of hearing loss are found in musicians than the ISO 1999 [8] predicts [4, 9–15]. Female musicians heard equally well as their male counterparts of the same age group [11, 16]. Several authors reported a better hearing of the right ear compared to the left ear [5, 14, 16, 17]. Especially with violinists, the volume of their own instrument is a dominant factor [18]. The left ear near the instrument is averagely exposed to 4.6 dB more than the right ear, which is averted from the instrument. In a literature study on the sonic load and hearing of musicians Sataloff [19] comes to the conclusion that musicians can produce dangerous sound levels, but further investigations would be necessary with regard to the effect on the hearing. A German study [20] to the hearing status of orchestral musicians found more than 50% musicians with hearing loss ≥15 dB and equivalent sound exposure levels L_EX_ > 90 dB(A).

**Fig. 1:**
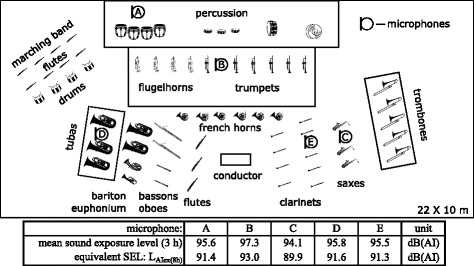
Military musicians at a three hours rehearsal with sound measurements at 5 microphone positions **a**-**e**. Mean sound exposure levels within 3 h and recalculated Levels to one working shift: L_AIex(8h)_ in dB(AI). (Illustration modified from F. Pfander, 1985) [22]

Overall, there is a rather heterogeneous picture regarding the hearing of musicians in the literature, which presents partly contradictory results and is not always comparable in terms of methodology. An important step towards standardization could be the application of a new parametric method by Bo et al. (2016) for the noise risk assessment of professional orchestral musicians [21]. The present longitudinal study examines military musicians who have been audited twice at intervals of more than 13 years. As the first examination [22] was carried out, sound measurements were also taken during rehearsals and concerts. A typical rehearsal with the musicians in a rehearsal room with corresponding sound measurements is shown in Fig. 1.

All microphone positions, with values up to 93 dB (A), show equivalent sound exposure well above the upper exposure action values [6].

The comparison of two audiograms to the same persons should enable to make reliable statements about the development of hearing or the development of a hearing damage in these persons under sound exposure far above the upper exposure action values [6].

## Methods

### Participants

Out of a total of 58 male military musicians of the army music corps in Bremerhaven 39 musicians could be tested a second time after about 13 years. Only complete data sets were evaluated. Three musicians who suffered from a cold were excluded. A total of 36 musicians could be evaluated in the present longitudinal study. Existing hearing damage was not an exclusion criterion, as only the development of hearing was of interest, whilst comparing the two hearing tests.

### Instrumentation

For the audiometry 4 audiometers of the company Hortmann type CA540 and an audiometer of the company Maico type MA53 in connection with circum-aural transducers type HDA200 of the company Sennheiser were used. These transducers were calibrated according to ISO 389–8 [23] and ISO 389–5 [24]. Hearing tests were performed with pulsed sine tones. The data was displayed and stored with the software Avantgarde 2.0 of the company Nuess via the serial interface RS 232 in PCs.

The first audiometry tests were performed with audiometers from Siemens type T31 and Beomat 2005 SR with supra-aural transducers from Beyer type DT 48 with sinusoidal tones. The tranducers were calibrated according to ISO 389–1 [25]. The audiometers were not yet connected to computers, but the hearing thresholds were entered on cardboard forms of the manufacturers. For the evaluation, the threshold values were transferred individually into a database.

### Acoustic measurements

The acoustic measurements during the rehearsal were carried out by the technicians of Pfander [22]. The measurements were performed with three ½ inch free-field microphones type 4165 (at A, B and C) and two ½ inch free-field microphones type 4133 (at D and E). All with preamps type 2619 and amplifier type 2606 of Brüel & Kjær (Danmark). The measure points A, B and C were recorded with a level statistics device type 4426 of Brüel & Kjær, and the points D and E with a PCM-recorder combination PCM-F1/SL-F1E from the company Sony (Japan).

### Software und statistics

The data processing was carried out with the Office programs Access and Excel Microsoft Office 2010, whereby even simple T-tests were used. The age-related hearing loss from ISO 7029 (2nd and 3rd editions) [24, 25] was also calculated in Excel and included for comparison. A repetitive multivariate ANOVA was performed using SPSS 15 with 3 inner-subjects factors (repeat) audiogram, frequency and ear and 2 between-subjects factors instrument and acoustic trauma. The statistical evaluation addressed the frequencies 3, 4 and 6 kHz, in which all datasets were complete. Only at 10 kHz three musicians had missing data values. One of these musicians had no value on both ears and two only on the left ear.

## Results

### Hearing thresholds

At intervals of about 13.3 years (mean age of the groups 31.4 and 44.7 years) military musicians of the German Federal Armed Forces were checked twice by means of audiometry and questioning.

**Fig. 2:**
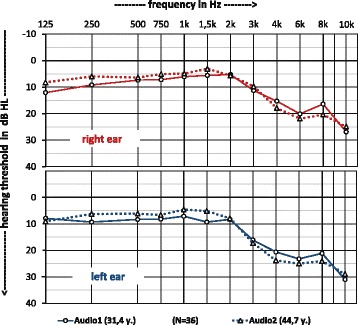
Repeated measured mean hearing thresholds of military musicians at both ears. Time difference is 13.3 years

**Fig. 3:**
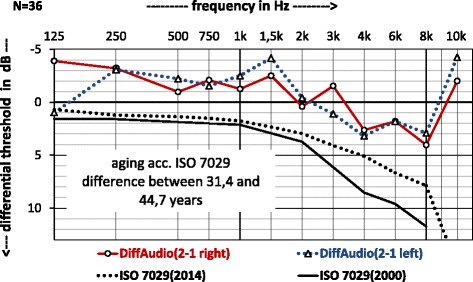
Audiograms (difference between later audiogram (2) and first audiogram (1)) of military musicians taken within a 13.3 years time-span, thereby comparing both ears separately with the age-induced hearing loss according to the current ISO 7029:2000 [26] and the draft ISO 7029:2014 [27] at the same mean ages

In Fig. 2 the average hearing ability of the musicians for both the right and left ear as well as at the time of the first measurement (Audio1) and 2nd measurement (Audio2) is shown separately. With the exception of the frequency at 125 Hz in the left ear, a hearing improvement was observed in both ears over all frequencies below 2 kHz over time, ranging from 1 to 4 dB. At 2 kHz, on average there was no change. At 3 kHz, the hearing thresholds behave differently in both ears. Over time, the right ear has improved by about 1 dB while the left ear has deteriorated by about 1 dB. Between 4 and 8 kHz the later audiometry shows a 2 to 5 dB worse hearing than the first examination. At 10 kHz, a slight improvement in hearing thresholds can be observed. Overall, the hearing of the right ear is slightly better than the left, which is shown in Fig. 3.

Figure 3 shows the averaged differences in auditory thresholds (Audio 2 minus Audio 1) for left and right ear seperately. These differences were compared to the current standard on hearing ISO 7029:2000 [26] and also to the new draft ISO 7029:2014 [27] for the age group between 31.4 and 44.7 years in the same diagram. It can be seen from this, that there are clear deviations between the expected and the measured hearing losses. Based on the new ISO standard (dashed black line in Fig. 3), the expected hearing loss is lower than for the still valid ISO from the year 2000 (solid black line in Fig. 3). The hearing loss differences in military musicians between both measurements across all frequencies is similar to the difference according to ISO aging. Almost across all frequencies, the military musicians show less age impaired hearing compared to the predicted aging according to the ISO standards with a deviation of about − 5 dB, which corresponds to an entire setting level in the audiometer. At 10 kHz, there is a slight improvement in the hearing thresholds of − 2 dB at the right ear and − 4 dB at the left ear. While ISO 7029:2000 [26] has no standard values at 10 kHz, the provisional new ISO standard (2014) [27] for this frequency predicts a hearing loss of 15.6 dB between the ages of 33.4 and 44.7 years. The difference in hearing thresholds between the two ears varies between 0 and 5 dB, with the curves crossing several times. Positive values indicate a deterioration in hearing.

**Fig. 4:**
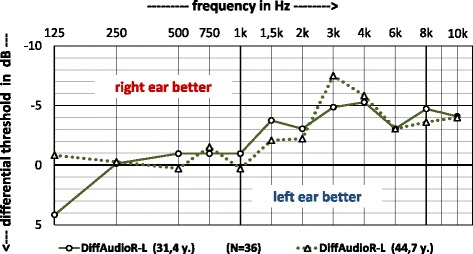
Audiograms (difference between right and left ear) of military musicians taken within a 13.3 years time-span. Negative values show better hearing of the right ear and positive values of the left ear

The average differences between the hearing thresholds of the right and left ear are shown in Fig. 4. It can be seen that the asymmetry of the ears at frequencies below 1.5 kHz is in a range between 0.2 and − 1 dB. A clear exception is seen at 125 Hz (in the first audiometry), where the hearing of the right ear in comparison to the other is 4 dB worse than that of the left ear. By subtracting the average hearing thresholds, the age-related, but also the noise-induced threshold differences are excluded, provided the effect is assumed to be the same for both ears. However, this does not seem to apply especially in the frequency range above 1 kHz. Between 2 and 7.5 dB we observe better hearing thresholds in the right ear. Averaged for 3, 4 and 6 kHz, the differences are about 5 dB. That this is a real effect is shown in the statistical significance for the ear factor at *p* = 0.036. A clear trend towards larger deviations at higher frequencies can be seen.

### Statistics

The statistical verification of relationships was performed with a multifactorial analysis of variance (ANOVA) with repeated measurements based on 5 factors and their interactions. Three factors: the frequency with three measurement levels (3, 4 and 6 kHz, which are most sensitive to high sound levels!), The ear (right / left) and the audiogram (first and second) are the inner-subjects factors (repeated measures) and two factors: the musician’s instrument (*N* = 14) and the history of the experienced acoustic shocks, both mentioned in the questionnaire, represent between-subjects factors.

Significant differences were only found for the within-subjects factors “frequency” and the “ear”, while the auditory thresholds of audiograms 1 and 2 did not differ significantly for a time span of about 13 years. The *between subject* factor “instrument” with 7 trumpets, 6 clarinets, 5 trombones, 4 horns, 3 percussionists, 2 tubes and saxophones each and 1 baritone, conductor, electric bass, bassoon, transverse flute, keyboard and piano did not show any significant differences in the hearing thresholds (*p* = 0.292 as seen in Table 1). Also, the acoustic shocks mentioned in the questionnaire had no significant impact. All interactions of the individual factors were also not significant, as shown in Table 1, even those with the significant factors of frequency and ear. More interactions were calculated than indicated in Table 1, all of which were not significant.

**Table 1:**
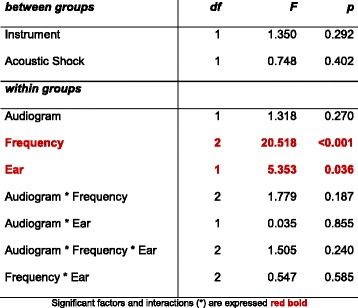
Results of a multifactorial analysis of variance (ANOVA) with repeated measurements. Two between-subjects factors: instrument and acoustic shocks and three within-groups factors: audiogram (first and second), frequency (3, 4 and 6 kHz) and the ear (right/left) with four interactions

### Noise induced hearing loss

With the equation: N50 = [u + v * lg (t / t0)] * (L_EX,8h_ - L_0_)^2^ from the ISO 1999 [8] the median of a hearing loss can be predicted. Here, u, v and L_0_ are frequency-dependent table values of ISO 1999 [8]: **t** is the duration of the load, **t**_**0**_ 1 year and **L**_**EX,8h**_ the determined average sound load during the time period **t**.

The duration of sound exposure *t* = 13.3 years. The average sound load L_EX,8h_ = 91.4 dB(A) as well as determined at microphone position A (See Fig. 1).

This results in predicted hearing losses for the frequencies 3 kHz: 11.2 dB, for 4 kHz: 14.4 dB and for 6 kHz: 9.6 dB. These hearing losses are a rough estimate and are based on the assumption that the sonic load of 91.4 dB(A) persisted for each musician and every working day during the 13.3 years without using ear protection devices or any other protective measures.

## Discussion

When comparing the two studies with an average interval of 13.3 years in Fig. 2, first of all the great similarity of the average audiograms stands out. At all frequencies below 2 kHz with the exception of 125 Hz in the left ear, the second audiometry is even better than the first examination. At 2 kHz, the hearing thresholds of both examinations are almost identical. The frequency at 3 kHz shows a different behaviour for both ears. For the right ear, the second audiogram is 1 dB better and for the left ear the first one is. At 4, 6, and 8 kHz frequencies, the auditory curves are parallel, with the auditory thresholds being better for the first audiogram than the second, as might be expected. Together with the 3 kHz frequency, this range is the most sensitive to the harmful effects of noise. The hearing threshold at 10 kHz behaves contrary to expectations. Although the presbyacusis is most pronounced at high frequencies, better thresholds are seen in the later study of military musicians. For clarification, these differences in the threshold values in Fig. 3 were highlighted and compared with the predicted age-impairment from ISO 7029 (2nd and 3rd editions) [26, 27]. The curves all have a similar course over the frequencies, but the ISO curves are clearly below the average threshold differences obtained from the measurements in the later and in the first audiogram (see legend to Fig. 2 called “DiffAudio”). These differences are greatest between the observed and the by ISO predicted thresholds at 10 kHz. Here, − 3 dB (both ears combined) contrasts with a value of 15.6 dB (ISO 7029:2014) [27]. The difference is therefore 18.6 dB and shows the opposite of a presbyacusis.

Musicians in classical orchestras (Chicago Symphony) [15] have high sound pressure levels with a mean of 90 dB(A) created by their instruments while practicing their profession for 15 h per week. Converted to a 40 h shift per week the mean levels are 85.5 dB(A). Impulse peaks reaches 143.5 dB, above the upper exposure action value of 137 dB(C) [6].

Finnish members of the National Opera are exposed to sound levels above the upper action exposure values of 85 dB (A) [28].

In an Australian study [29] mean L_EX_ levels for the brass section could be measured up to 90.7 dB(A) and peak levels for the percussion section reached 146,9 dB(C).

In a Canadian study [1], the average sound levels of wind players were between 88 dB(A) (oboe) and 94 dB(A) (trumpet) during 3 h. In comparison workers were exposed to industrial noise for about 2000 h per year, musicians for about 360 h per year. Thereby a correction value of − 7.4 dB was calculated and used.

In his measurements (Fig. 1) Pfander [22] uses the time constant “impulse” because of the high peak values, which occured especially in drums. Thus, the measured values are no longer directly comparable to the literature values. However, the burdens of military musicians may actually be higher than those of classical orchestra musicians, because of the over-proportionally large brass-section in comparison to the classical orchestra composition.

Nevertheless, the hearing thresholds at 3, 4 and 6 kHz with *p* = 0.270 (audiogram) are not significantly different between the two examinations over 13.3 years of exposure. ISO 1999 [8] predicts noise-induced permanent threshold shift (NIPTS) for sound levels of 91.4 dB in 13.3 years of 11.2 dB (3 kHz), 14.4 dB (4 kHz) and 9.6 dB (6 kHz). This will by far not be confirmed by the second investigation: Here we observed neither hearing loss (positive threshold values in Fig. 3) at 3 kHz is ±1 dB, at 4 kHz 3 dB and 2 dB at 6 kHz, NIPTS (ISO 1999) [8] nor presbyacusis (ISO 7029) [26, 27]. The difference between expected and observed value is about 10 dB. In order to confirm the measured values by the ISO 1999 [8], average sound levels of about 83 dB would have to be assumed in the calculation.

In addition to the music played during working hours, additional exposure is added by playing music during leisure time and other noisy hobbies [20]. Further exposure is due to impulse noise in the regular military shooting practice. The dip in the audiogram between 2 and 8 kHz also shows these effects of the impulse noise. In the questionnaire, 13 musicians stated that they had suffered an acoustic trauma during their time as soldiers. There is no indication from the audiogram of 8 musicians, so that the temporary hearing loss has receded. 5 musicians with the indication of an acoustic trauma had a corresponding sink in the audiogram. On the other hand, a further 12 musicians showed clear traces of past hearing damage due to impulse noise in the audiogram, without having reported this event in the questionnaire. In two cases, the question of acoustic trauma was not answered. In conclusion, less than one third (*N* = 5) of all observed/suspected acoustic traumata (*N* = 17) are identified by the survey. Therefore, the missing statistically significant correlation of the hearing thresholds with acoustic trauma (yes/no) (*p* = 0.402) can be explained. Sonic events that lead to clearly visible sinks in the audiogram are usually kept in mind by those affected, especially since their consequences can have an impact on the performance of the musicians. The discrepancy between the anamnestic questionnaire and the audiometric findings on acoustic trauma may well be the result.

The frequency-dependent differences at 3, 4 and 6 kHz, which statistically describe the observed drop in the hearing curve are highly significant (*p* < 0.001). Since the thresholds at 8 kHz are better than at 6 kHz, we can speak of a sink in the audiogram indicating a history of noise exposure.

In the current investigations, a significantly better hearing of the right than the left ear was found (see Table 1 and Fig. 3). That this is a real effect is shown in the statistical significance for the ear factor at *p* = 0.036.

This difference may be due to a generally greater vulnerability of the left ear in comparison to the right ear [30] or to an asymmetric impact of damaging sound [18, 22]. The latter would also be an indication of the origin of the hearing damage caused by impulse or shooting noise and especially not by the practice of music. Except for the flutes and horns most of the wind instruments radiate the sound symmetrically forward or upward.

he factor instrument also was not significant (*p* = 0.292), which is surprising in view of the large differences between the instruments in terms of maximum volume and their frequency spectrum. In the statistical review, the factor instrument had no significant influence nor possible interactions with the ear (not listed in Table 1). As early as 1985, Johnsson et al. [31] found that the instrument being played and the position in the orchestra did not lead to significant differences in hearing, which is confirmed by the current data.

Possible reasons for only small differences in the two investigations could be the different measurement techniques and the use of pulsed test tones in the second study with better measurement results, compared to continuous tones in the first study. However, there is no evidence in the literature, on the contrary, only marginal differences were found [32]. Similarly, the circumaural transducers of the second study have a better external noise attenuation than the supra-aural transducers of the first study and measurement results are more reliable [33]. Another limitation may be the relatively small number of 36 participants, which, however, is of less importance in longitudinal than in cross-sectional studies.

## Conclusions

Overall, the musician’s hearing has remained largely unchanged during the period under review, just over 13 years. The effect of noise on hearing remains far behind the predictions of ISO 1999 [8]. Similarly, the aging predicted by the ISO 7029 [26, 27] with progressive lowering of the thresholds in the collective of military musicians cannot be confirmed. In this sense we can speak of a delayed presbyacusis in musicians. This could be due to the highly selected collective of musicians with special training in hearing. By adaptation processes the practice of music with planned sound effect does not seem to be as damaging to the hearing due to the stapedius reflex, as unwanted pulsed industrial noise with the same sound levels. But this should not lead musicians to negligently deal with their hearing, but to ensure that special peak noise levels are avoided or mitigated with ear protection and to ensure adequate recovery of hearing during break-times.
